# Establishment of human metastatic colorectal cancer model in rabbit liver: A pilot study

**DOI:** 10.1371/journal.pone.0177212

**Published:** 2017-05-05

**Authors:** Veronica Prieto, Johannes M. Ludwig, Alton B. Farris, Ganji Purnachandra Nagaraju, Taoreed O. Lawal, Bassel El-Rayes, Hyun S. Kim

**Affiliations:** 1 Division of Interventional Radiology and Image-guided Medicine, Department of Radiology and Imaging Sciences, Emory University School of Medicine, Atlanta, GA, United States of America; 2 Division of Interventional Radiology, Department of Diagnostic Radiology, Yale University School of Medicine, New Haven, CT, United States of America; 3 Department of Pathology and Laboratory Medicine, Emory University School of Medicine, Atlanta, GA, United States of America; 4 Departments of Hematology and Medical Oncology, Emory University School of Medicine, Atlanta, GA, United States of America; 5 Yale Cancer Center, Yale School of Medicine, New Haven, CT, United States of America; Northwestern University Feinberg School of Medicine, UNITED STATES

## Abstract

**Rationale and objectives:**

To develop a human metastatic colorectal cancer (mCRC) model in a rabbit liver.

**Materials and methods:**

Immunosuppression in 4 adult New Zealand White rabbits weighing 3.5 to 4.5 kg was induced with daily subcutaneous injection of 15 mg/kg Cyclosporine A (CsA). On day 3 open mini-laparotomy was performed and 0.2 ml (1.8x10^5^ cells) suspension of HCT-116 and HT-29 human CRC cells were injected into the left and right medial lobe respectively. On day 10 the CsA dose was reduced to 10 mg/kg daily maintenance dose. Rabbits were weighed weekly, closely monitored for CsA side effects (weight loss, gingival hyperplasia and gut modification). Rabbits were sacrificed 5, 6, 7, and 8 weeks after cells injection. Liver tumors were collected for histopathology and immunohistochemical analysis.

**Results:**

HT-29 Tumor growth was observed in 3 rabbits (75%). Tumors measured 3, 4 and 6 mm after 5, 6 and 8 weeks respectively. Microscopically, tumors contained hyperchromatic, pleomorphic cells that stained for monoclonal carcinoembryonic antigen (CEA), polyclonal CEA, cytokeratin 20, vascular markers (CD31, CD34), and vascular endothelial growth factor (VEGF) by immunohistochemistry, supporting involvement by the poorly differentiated HT-29 colorectal cancer cell line. No gross tumor growth or microscopic viability was observed from HCT-116 cell injection. CsA extra-hepatic manifestations included minimal gum hyperplasia and decrease in gut motility in 3 rabbits (75%), which was treated with Azithromycin 15 mg/kg and Cisapride 0.5 mg/kg every 12 hours, respectively.

**Conclusion:**

We successfully developed a human metastatic colon cancer model in immunosuppressed rabbit liver using HT-29 cells.

## Introduction

Complete hepatic tumor resection offers the best chance for long-term survival of patients with colorectal carcinoma (CRC) liver metastases [1–3]. In addition to standard systemic chemotherapy, locoregional therapies including transarterial approaches and ablative modalities may be amenable for treatment of liver metastases not suitable for resection [4].

For the evaluation of experimental locoregional therapies of liver cancer, the rabbit VX2 tumor allograft model has played a longstanding and important role due to its manifold advantages including predominant hepatic artery blood supply, rapid tumor establishment, suitability for imaging, and the possibility to access the hepatic artery specifically via intra-arterial catheters for the selective administration of experimental drugs [5–8].

However, VX2 carcinoma originally derived from skin cancer of cottontail rabbits caused by a papilloma virus infection [9, 10]. Histopathologically, the VX2 carcinoma is an anaplastic squamous cell carcinoma which may not be an ideal CRC liver metastasis model for the evaluation of experimental CRC treatment approaches limiting transfer of findings to humans [11–13]. Furthermore, VX2 tumor growth is associated with a high rate of central necrosis which can impede the evaluation of the actual treatment induced tumor necrosis. Also, the VX2 tumor relies on an *in vivo* culturing in e.g. the hind legs of a rabbit since *in vitro* growth has been proven insufficient [14–16].

Xenograft mouse and rat animal models have been established using human colorectal cancer cell lines providing insight into the biological actions and might establish a molecular basis for the development of systemic new cancer therapeutic agents [17–19]. In these models however, intra-arterial approaches via the groin artery are not feasible to perform or have only a limited applicability and versatility due to the small animal and vessel size. We therefore developed a novel rabbit liver metastatic human CRC model well-suited for the evaluation of novel intra-arterial treatment approaches.

## Materials and methods

### Animals

This study was approved by the Animal Care and Use Committee of Emory University (Permit Number: 2001192) and all animal care and procedures were performed under institutional guidelines and have been performed in accordance with the ethical standards as laid down in the 1964 Declaration of Helsinki and its later amendments. Surgery was performed under general anesthesia and all efforts were made to minimize suffering. All male adult New Zealand White rabbits weighing 3.5 to 4.5 kg were provided by Charles River Laboratories International, Inc., St-Constant, Quebec, Canada. A total of 4 rabbits were used in this exploratory pilot study.

#### Immunosuppressant therapy

Immunosuppression was performed similar to previous descriptions [20–23]. Cyclosporine A (CsA) (Leiter’s Pharmacy, San Jose, CA), stock concentration of 50 mg/ml diluted in 0.9% sodium chloride, was used to induce immunosuppression through sub-cutaneous injection in the dorsal neck region of the rabbit. The dosage schedule was in a dose of 15 mg/kg per day for 3 days before liver injection and for 1 week thereafter. To further reduce CsA associated toxicity, the dosage was reduced to 10 mg/kg per day for the last 7 weeks of the experiment. CsA doses were adjusted weekly according to each animal’s body weight. CsA administration was maintained throughout the life of the animal to prevent spontaneous tumor regression and animals were monitored daily for signs of CsA toxicity such as gingival hypertrophy, drooling, diarrhea, and weight loss. Conditions such as minimal gum hyperplasia and decrease in gut motility were treated with Azithromycin 15–20 mg/kg by mouth once a day for 7 days and Cisapride 0.5 mg/kg by mouth every 12 hours, respectively.

### Human cancer cell lines

Two types of human cancer cells of metastatic colorectal carcinoma (mCRC) were used; human colon carcinoma HCT-116 and human colon adenocarcinoma HT-29 cell lines from American Type Culture Collection (ATCC), Manassas, VA. Following the provider specifications, the cell lines were thawed and subcultured. After cell growth, a suspension of 1.8x10^5^ cells/0.2 ml of HCT-116 and HT-29 were separately prepared and placed into a 1 ml luer lock syringe on ice until injection. To decrease the possibility of leakage slow injections were done for two minutes, followed by finger compression for 2–3 minutes.

### Implantation of HCT-116 and HT-29 into liver

**Fig 1:**
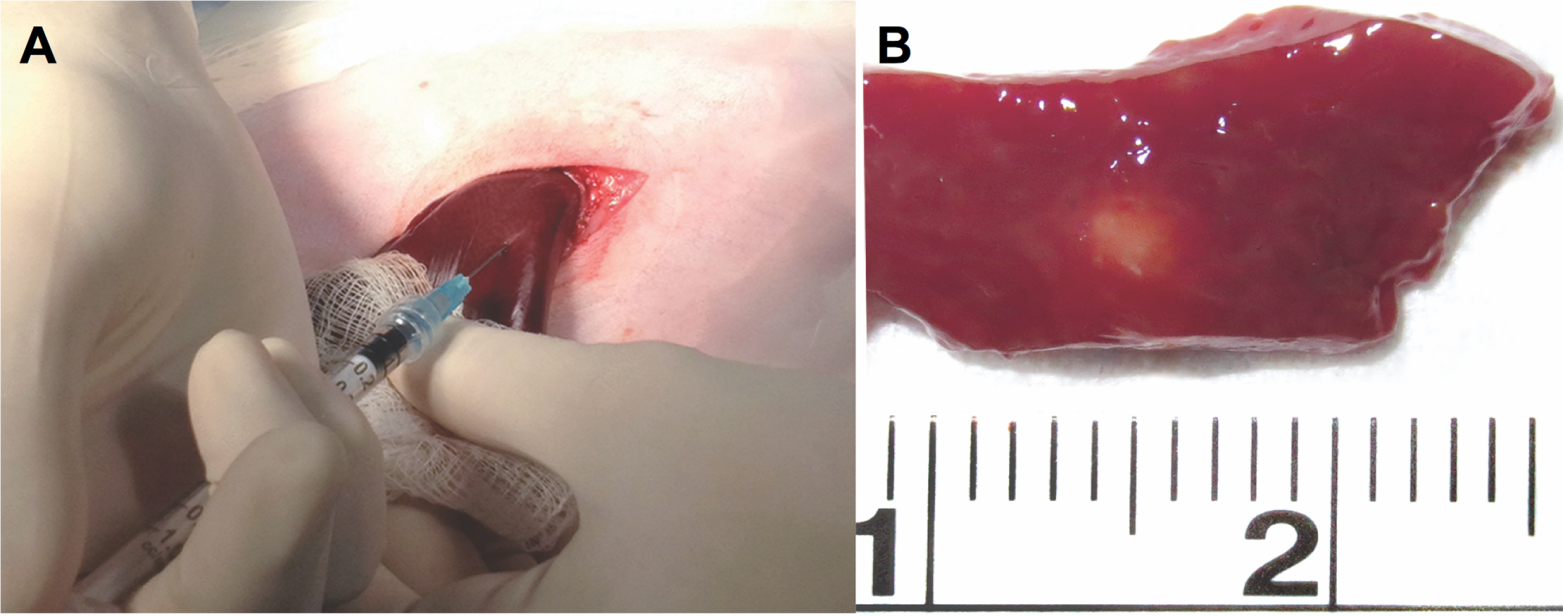
Tumor implantation and explanted tumor. (A) In vivo rabbit liver (right medial lobe) showing mCRC cell line injection technique. (B) Sectioned specimen shows (arrows) mCRC tumor (0.3 cm) after 5 weeks of tumor growth.

Rabbits were pre-anesthetized with an intramuscular mixture of 3 mg/kg xylazine HCL and 35 mg/kg ketamine hydrochloride. After approximately 15 minutes, IV access was established via a marginal ear vein and isoflurane inhalation was provided to maintain a surgical plan for anesthesia. The abdomen of each recipient rabbit was shaved and disinfected with ethanol and povidine iodine solution. Standard sub-xyphoid mini–laparotomy (2–3”) was performed. The liver of each rabbit was exposed and a suspension of 1.8x10^5^ cells of HCT-116 in a volume of 0.2 ml was slowly injected into the liver parenchyma of the medial left lobes, using a 1 ml luer lock syringe and 25G x 5/8″ needle. A 0.2 ml suspension of 1.8x10^5^ cells of HT-29 was injected into the liver parenchyma of the medial right lobes, using the same technique (Fig 1A). The medial lobes of the liver were selected because of easy access and their thickness, allowing them to tolerate the cell line injection [24]. Furthermore, the manipulation of these lobes back into the abdominal cavity requires less handling, thus reducing the possibility of cell leakage in addition to the gentle compression of puncture site for 2–3 minutes after needle removal. After confirming no bleeding or spillage of tumor cells the liver was then repositioned back to its original intra-abdominal space under aseptic technique. The abdominal incision was then closed in layers. After surgery, the animals were placed in cages and monitored in the animal laboratory under close and direct supervision of a physician or technician until they recovered from anesthesia. Postoperatively, analgesic buprenorphine (0.02–0.05 mg/kg) was injected intramuscularly. The tumors were allowed to grow for up to eight weeks. Daily monitoring for signs and symptoms of CsA side effects such as mood changes (lethargy), change in eating (loss of appetite), body weight loss, gum hyperplasia, gut motility modification (size, volume, consistency of stools), liver failure (sleepy, swollen abdomen), kidney failure (decrease or absence of urine), and post-surgery complications, was performed for the entire 8 week study interval.

### Necropsy and histopathology

Following pre-sedation with the above described mixture, a single rabbit was randomly chosen for sacrifice at four different time points (5, 6, 7 and 8 weeks post liver inoculation) using an intra-cardiac injection of 150–200 mg/kg sodium pentobarbital (Euthasol; DV Medical Supply, Redondo Beach, CA). All animals underwent necropsy to determine tumor growth in the liver (Fig 1B). Immunohistochemical staining was performed using Dako automated benchmark stainers (Dako Group, Carpinteria, CA) in the two largest tumor specimen to support the colorectal carcinoma origin of the malignant cells. Antibodies applied for confirmation of tumor presence included cytokeratin 20 (monoclonal clone Ks20.8, titration 1:40, Dako), monoclonal carcinoembryonic antigen (CEA, clone: B01-94-11M, titration 1:200, Biogenex, San Ramon, CA), polyclonal carcinoebryonic antigen (titration 1:16,000, Dako). Microvessel density and neoangiogenesis were evaluated by staining for CD31 (monoclonal JC70A clone; Titration: 1:80; Dako Corporation, Carpinteria, CA), CD34 (monoclonal QBEND clone; Titration: 1:320; Dako), and vascular endothelial growth factor (VEGF; titration 1:80; Santa Cruz Biotechnology, Inc., Santa Cruz, CA) [25, 26].

## Results

### Tumor growth

**Fig 2:**
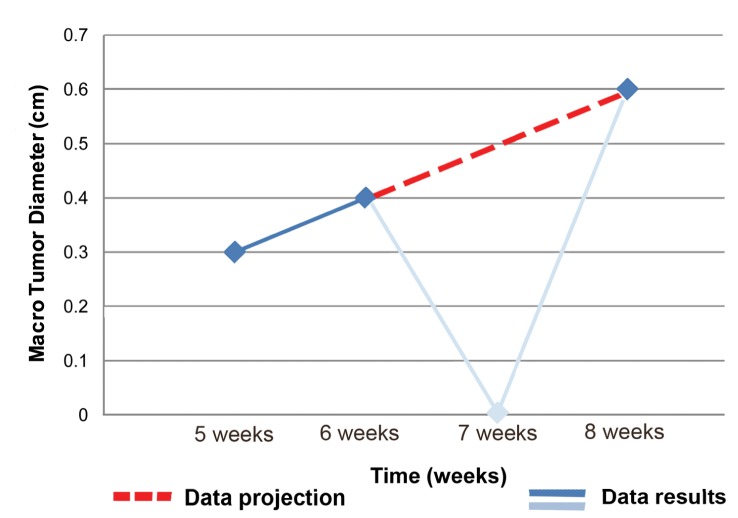
Tumor growth. Tumor growth progression (in cm) of the human colorectal carcinoma xenograft HT-29 over time. Note: The continuous line shows the observed tumor growth progression whereas the discontinuous illustrates the expected growth projection in case of successful tumor growth.

Successful tumor growth was observed in the right medial lobes of 3 rabbits with a successful implantation rate of 75% using HT-29 cells. Tumor sizes of 0.3 cm, 0.4 cm and 0.6 cm at 5, 6 and 8 week endpoints were observed, respectively (Fig 2). No viable tumors were found in the left medial lobes from HCT-116 cell injections (successful implantation rate 0%) (Table 1). No extrahepatic metastases were evident in necropsy at all time points.

**Table 1:** Overview of the intrahepatic tumor growth. Overview of the intrahepatic tumor growth of the human metastatic colorectal carcinoma (mCRC) cell lines HT-29 and HCT-116 post injection.

Colorectal Carcinoma Cell Line	Cells injected	Sacrifice (weeks)	Tumor Diameter (cm)	Tumor Growth
HT-29	1.8 x 10^5^	5	0.3	**Positive**
		6	0.4	**Positive**
		7	0	Negative
		8	0.6	**Positive**
HCT-116	1.8 x 10^5^	5	0	Negative
		6	0	Negative
		7	0	Negative
		8	0	Negative

### Histopathological evaluation of tumor implants

**Fig 3:**
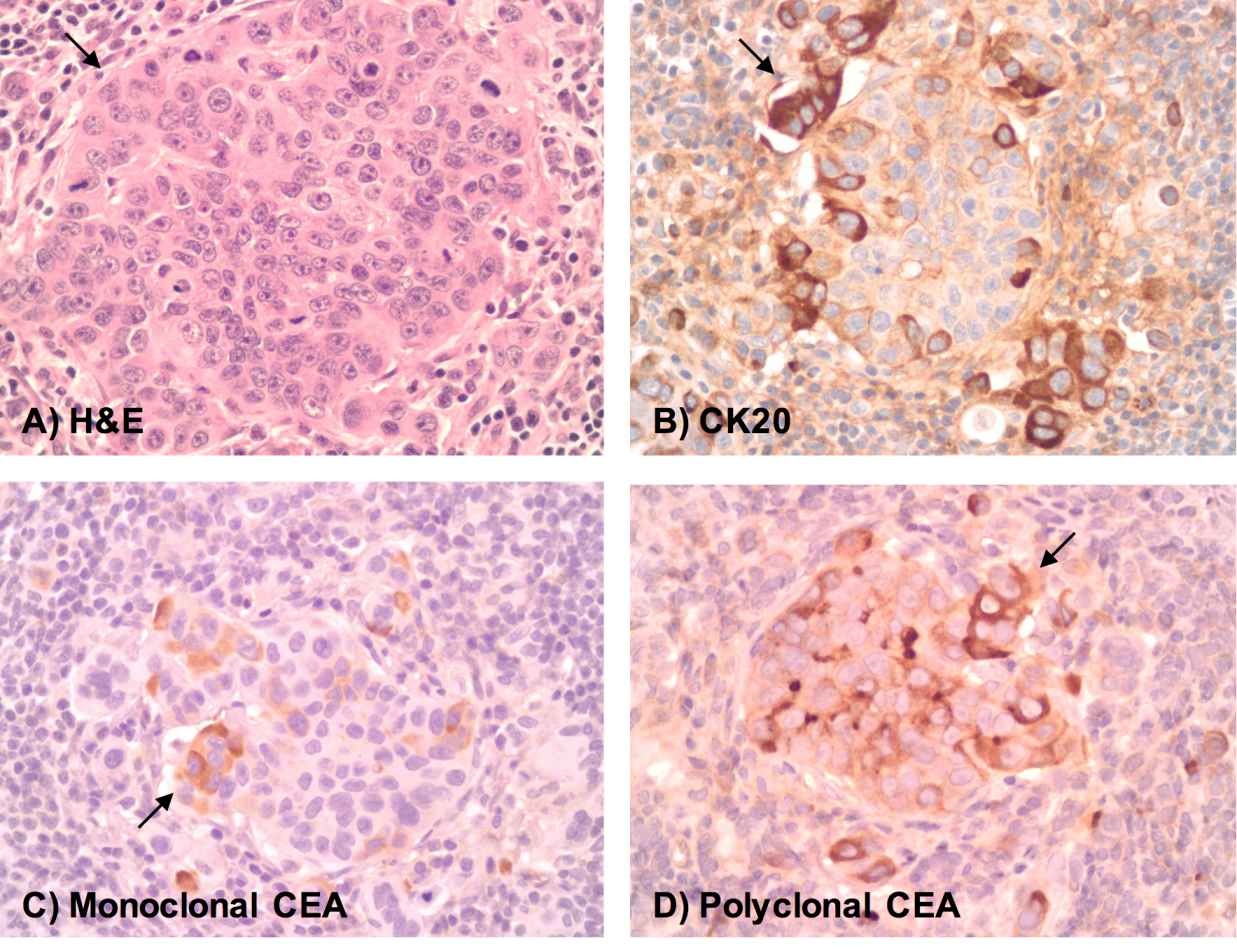
Hematoxylin & Eosin (H&E) and immunohistochemistry staining of the mCRC HT-29 tumor xenograft. A) Tumor cells (arrow) present in a cluster of pleomorphic, hyperchromatic cells (H&E). Immunohistochemistry shows that the tumor cells stain positively (arrow) for Cytokeratin 20 (B) monoclonal carcinoembryonic antigen (CEA) (C), and polyclonal CEA (D). All images are shown at an original magnification of 400x.

**Fig 4:**
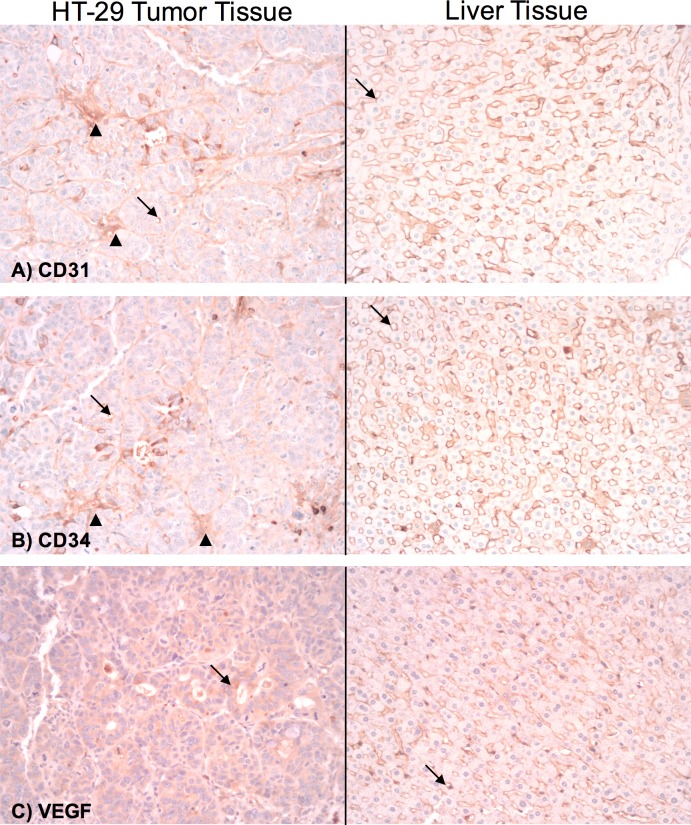
Immunohistochemistry of angiogenesis markers. The explanted HT-29 tumor xenograft on the left and normal liver tissue on the right were stained for CD31 (A), CD34 (B), and vascular endothelial growth factor (VEGF) (C). For CD31 and CD34, the microvessel endothelium was highlighted in both the tumor and in normal liver sinusoids [arrows]. The liver tumors had staining with the endothelial markers CD31 and CD34 at a lower density than the normal liver hepatic sinusoids. Stroma between tumor cell clusters stained with CD31 and CD34 [arrowheads], suggesting an induction of angiogenesis. VEGF showed background staining in the tumor [arrow] at a higher level than normal liver tissue, suggesting cytoplasmic induction of VEGF expression in an attempt by the tumor to induce angiogenesis. Sinusoids in the normal liver tissue stained with VEGF [arrow]. All images are shown at an original magnification of 200x.

Microscopically, tumors were confirmed to contain hyperchromatic, pleomorphic cells surrounded by fibrous stroma and chronic inflammation. Some cells were present in circumscribed clusters suspicious for lymphovascular invasion, while others exhibited single cell infiltration of fibrotic desmoplastic-type stroma. Only scattered small focal necrosis was evident in tumor explants. Immunohistochemistry showed that the tumor cells stained positively for cytokeratin (CK) 20, monoclonal and polyclonal carcinoembryonic antigen (CEA) (Fig 3). These morphologic and immunohistochemical findings confirmed involvement by the poorly differentiated colorectal cancer cell line. Furthermore, immunohistopathological evaluation of neoangiogenesis was evaluated in explanted HT-29 tumor xenografts and normal liver tissue for direct comparison (Fig 4). For CD31 and CD34, the microvessel endothelium was highlighted in both the tumor and in normal liver sinusoids. The liver tumors were positive for staining with the endothelial markers CD31 and CD34 at a lower density than the normal liver hepatic sinusoids. Stroma between tumor cell clusters stained with CD31 and CD34 suggesting an induction of angiogenesis (Fig 4A & 4B). VEGF showed background staining in the tumor at a higher level than normal liver tissue, suggesting cytoplasmic induction of VEGF expression in an attempt by the tumor to induce angiogenesis (Fig 4C).

### Safety and adverse events

All 4 rabbits tolerated the cell line inoculations. No leakage or bleeding was observed after cell line injections. Minimal gum hyperplasia in 1 rabbit at 2 weeks and in 2 rabbits at 5 weeks was observed as a consequence of the immunosuppressant therapy with CsA. Also, decrease in gut motility was observed in 3 rabbits. Total recovery of the animals was accomplished after treatment of those secondary effects of CsA with Azithromycin and Cisapride. No other secondary effects such as diarrhea, weight loss or further complications were recorded. No mortality was observed in between the time of tumor implantation and the sacrifice.

## Discussion

Colorectal cancer is the third most common cause of cancer related death with around 135.430 new cases and 50.260 expected deaths in the year 2017 in the USA. At time of diagnosis, up to 25% of patients present with distant metastases which increases to a total of 50% in the course of disease. Most of the patients with distant metastases cannot be treated curatively which currently limits the 5-year survival probability after metastasis diagnosis to around 9–17% compared to 88–93% in patients without any metastases [27, 28]. Currently, for patients with standard treatment refractory metastatic liver CRC, intra-arterial therapies allow for a median overall survival prolongation from ~6 months if untreated to 7.7–19 months [4, 29–31]. Since the survival outcome is still greatly lacking behind expectations, the development and evaluation of novel treatment approaches are highly warranted.

There are multiple studies that assess the use of human colorectal carcinoma cell lines, HT-29 [32] and HCT-116 [33, 34] in mice for biological, drug and imaging evaluation. However, these mice models are not appropriate for the use of most interventional techniques due to small animal size. Previous efforts to develop a human liver metastatic CRC model (HT-29) have also been undertaken successfully in immune incompetent rats [19]. Although liver tumor implants in rats theoretically allow for the evaluation of intra-arterial treatment approaches, it comes at the cost of a major and difficult to perform microscopic animal surgery involving a median laparotomy with abdominal cavity exposure and cannulation of the proper hepatic artery with a needle for drug injection [35, 36]. In rabbits on the other hand, vascular access can be gained with relatively minimal effort via the groin artery, similar to humans, which also allows for proper angiographic evaluation during the intervention. Moreover, the larger average caliber of the proper hepatic artery in rabbits (1.68 mm) compared to rats (0.29 mm) permits for the injection and evaluation of larger particles [25, 26]. However, the often used VX-2 cancer model in rabbits biologically differs from CRC limiting transferability of findings to humans [11–13]. We therefore developed a humanized CRC model in rabbits suitable for the evaluation of e.g. new drug or microspheres specifically designed for the treatment of colorectal carcinoma.

For the development we have chosen the HCT-116 and HT-29 cell lines due to their broad use in the evaluation of CRC anticancer drugs in current literature and their great availability for testing. Both cell lines are similar in nature in epithelial and adherent growth. It is a challenge to successfully use direct cellular injection into the liver parenchyma as the rate of tumoral cell leakage has been reported up to 50–60% [37, 38]. In our experiment, tumors were present in three of the four right lobes at the injection site with HT-29, with one failed case where it can be presumed that a leakage of injected cells or tumor regression due to insufficient immunosuppression occurred. There was no tumor growth using the HCT-116 cell line. This result can be potentially attributed to features of the particular cell lines, cell culture conditions, the preparation procedure, suppressing influence on HCT-116 cells by the injected HT-29 cell line or *in vivo* intrahepatic environmental factors, all of which warrant further investigation. Immunohistopathological evaluation of explanted HT-29 tumor specimen demonstrated an increased intratumoral neoangiogenesis induction in tumors (CD 31, CD34; Fig 4A & 4B) as well as an increased tumor cell VEGF expression compared to normal liver tissue. Thus, this model may be ideal for testing neoangiogenesis targeting therapies.

The observed signs of toxicity from the use of CsA to induce and maintain immunosuppression conditions as minimal gum hyperplasia and decrease in gut motility were successfully treated with Azithromycin and Cisapride. Diarrhea and weight loss were not observed. To avoid severe signs of CsA toxicity and the need to sacrifice study animals before the endpoint of our experiment, we lowered the dose of CsA to 10 mg/kg per day after one week of cell injections instead of maintaining 15 mg/kg per day for four weeks after inoculation, as shown in previous studies [20–23]. A sub-cutaneous injection to administer the CsA was used instead of the commonly utilized intramuscular approach. Because of this, the manifestation of these toxicity signs and the tumor growth in three rabbits suggested that the same level as intramuscular injections was reached and that the poor blood supply to subcutaneous tissue may not be an issue impeding blood concentration levels.

The development of human mCRC in a large animal model comes with numerous intrinsic challenges. Limitations of the present study include the small size of the tumor, which also restricted the evaluation of the tumor biology itself, lack of imaging and single time points for tumor explanation. The small sample size can also be seen as a limitation, although the purpose of this experiment was to achieve proof of concept. Moreover, implantation of two different cell lines in the same animal may have had an influence on tumor growth of each cell line. The potential to strengthen this model lies in future studies aimed at enhancing consistency of the model, tumor size as well as to evaluate the metastatic potential of this cancer model. Moreover, the cell lines utilized are of primary origin and may therefore not truly represent a metastatic CRC model. Although this is an immunosuppressant model that limits the evaluation of immunohistochemical biomarkers, our success and the results presented here are intended to stimulate further research in this area and provide insight into the significance of this preclinical human cancer model and applications.

In conclusion, we created a human metastatic colorectal cancer model in a rabbit liver with tumor growth in the liver using the HT-29 cell line.

## Abbreviations

CEA: carcinoembryonic antigen
CK: cytokeratin
CRC: colorectal cancer
CsA: cyclosporine A
HCT-116: human colorectal cancer cell line
HT-29: human colorectal cancer cell line
IV: intravenous
mCRC: metastatic colorectal cancer
VEGF: vascular endothelial growth factor
VX2: rabbit tumor cell line
